# A Comparative Pharmacokinetics Study of the Anti-Parkinsonian Drug Pramipexole

**DOI:** 10.3390/scipharm84040715

**Published:** 2016-11-18

**Authors:** Ratih S. I. Putri, Effi Setiawati, Syifa A. Aziswan, Fenny Ong, Raymond R. Tjandrawinata, Liana W. Susanto

**Affiliations:** 1 PT Equilab International Bioavailability and Bioequivalence Laboratory, Jl. RS Fatmawati Persil 33, 12430 Jakarta, Indonesia; ratih@equilab-int.com (R.S.I.P.); effi@equilab-int.com (E.S.); syifa.aziswan@equilab-int.com (S.A.A.); 2 Dexa Laboratories of Biomolecular Sciences (DLBS), Industri Selatan V Block PP No. 7, Jababeka Industrial Estate II, Cikarang, 17550 West Java, Indonesia; fenny@dexa-medica.com (F.O.); fenny@dexa-medica.com (L.W.S.)

**Keywords:** bioavailability, bioequivalence, Parkinson’s disease, pharmacokinetics, pramipexole

## Abstract

The present study aimed to compare pharmacokinetic parameters of two pramipexole 0.25 mg formulations in order to show bioequivalence. The study was conducted in a randomized, open-label, two-period, two-sequence, and crossover design, involving 23 healthy volunteers. One of the 0.25 mg formulations of pramipexole evaluated in the study was manufactured by PT Dexa Medica, Palembang, Indonesia, the other, used as the reference, by Boehringer Ingelheim Pharma GmbH & Co. KG, Ingelheim am Rhein, Germany. All eligible subjects were required to fast before each drug administration period, which was separated by a one-week washout period. Pramipexole concentrations in plasma were assayed using a validated ultra performance liquid chromatography with mass spectrometry (UPLC-MS/MS) detector. The evaluated pharmacokinetic parameters included the area under the plasma concentration curve from time zero to the last observed measurable concentration (AUC_0-t_), the area under the plasma concentration curve extrapolated to infinite time (AUC_0-∞_), the maximum plasma concentration (C_max_), the time to reach C_max_ (t_max_), and the plasma concentration half-life (t_1/2_). To evaluate the bioequivalence of those two pramipexole formulations, 90% confidence intervals (CIs) for geometric mean ratios of both formulations were calculated for AUC and C_max_ parameters, while t_max_ and t_1/2_ differences were analyzed on the non-transformed data using Wilcoxon matched-pairs and a Student’s paired *t*-test, respectively. The 90% CIs for the geometric mean ratios of the two pramipexole formulations were 95.89% (90.73%–101.34%), 95.53% (89.75%–101.68%), and 92.11% (84.35%–100.58%) for AUC_0-t_, AUC_0-∞_, and C_max_, respectively. There were no statistically significant differences for t_max_ and t_1/2_ between the two pramipexole formulations. It is concluded that two pramipexole formulations in this study were bioequivalent.

## 1. Introduction

Pramipexole is one of the most common dopamine-receptor agonist used for the symptomatic management of Parkinson’s disease (PD). The disease is a debilitating disorder of the nervous system and considered the second most common neurodegenerative disorder after Alzheimer’s disease [1,2].

It is suggested that a loss of neurons that produce dopamine, a chemical messenger in the brain, is responsible for many of the symptoms of PD [3]. Thus, the medication’s primary goal of this disease is to increase or substitute dopamine. Levodopa, the metabolic precursor of dopamine, is known as the first line therapy for PD. However, the administration of the drug for a long period can cause motor complications and dyskinesia. In addition, over time, the effect of the drug usually wears off [3,4]. One strategy to reduce that side effect and improve long-term outcome is to introduce symptomatic therapy with a dopamine-receptor agonist, such as pramipexole, and to add levodopa as a supplemental therapy when dopamine-receptor agonist monotherapy no longer provides adequate symptom control [5].

In the treatment of PD, the dose of pramipexole is increased gradually. The initial dosage of pramipexole hydrochloride, the usual form of pramipexole when given orally, is 125 µg three times daily in the first week. This is titrated to 250 µg three times daily in the second week and 500 µg three times daily in the third week. After that, the daily dose may be increased by 750 µg at weekly intervals to a maximum dose of 4.5 mg daily [5,6]. Pramipexole may also be used to treat restless legs syndrome (RLS), uncomfortable sensations in legs that commonly occur at rest and are usually relieved by moving the legs [7]. A single daily dose, 125 µg daily initially, 2 to 3 h before bedtime is given to remedy RLS, with a maximum dose of 750 µg daily [7].

The present study aimed to assess the pharmacokinetic profile of a 0.25 mg pramipexole formulation from PT Dexa Medica, Palembang, Indonesia, compared with that of a reference formulation from Boehringer Ingelheim Pharma GmbH & Co. KG, Ingelheim am Rhein, Germany, in order to demonstrate bioequivalence between the two.

## 2. Materials and Methods

### 2.1. Subjects

Eligible subjects were to meet the following inclusion criteria: healthy male or female subjects between 18 and 55 years old; non-smokers or light smokers, defined as smoking less than ten cigarettes per day; subjects with a body mass index within 18 to 25 kg/m^2^; and subjects with normal vital signs. This study excluded pregnant or lactating women, as well as those with any of the following: a family history of hypersensitivity or contraindication to pramipexole; any major illnesses in the past 90 days; any liver or renal dysfunction; positive test results for hepatitis B surface antigen (HBsAg), anti-hepatitis C virus (HCV), or anti-HIV; clinically significant hematology abnormalities; clinically significant electrocardiography (ECG) abnormalities; a history of anaphylaxis or angioedema; a history of drug or alcohol abuse; a history of any bleeding or coagulative disorders; and any surgical or medical condition that might significantly alter the pharmacokinetics of pramipexole. Subjects who took any concomitant medication, food supplement, or herbal medicine within 14 days before the study and participated in any clinical study within the past 90 days could not participate in the study.

This study was carried out in accordance with the Declaration of Helsinki [8], Good Clinical Practice [9], and Good Laboratory Practice [10]. Ethical approval from the Health Research Ethics Committee of the Faculty of Medicine, University of Indonesia (462/PT02.FK/ETIK/2010), was obtained before the conduct of the study. All subjects gave informed consent prior to their participation in the study.

### 2.2. Study Products

The test formulation in the study consisted of 0.25 mg pramipexole dihydrochloride monohydrate tablets produced by PT Dexa Medica (batch number K-10165-F-PSC-5), and the reference formulation consisted of 0.25 mg pramipexole dihydrochloride monohydrate tablets produced by Boehringer Ingelheim Pharma GmbH & Co. KG (Sifrol^®^, batch number 307338).

### 2.3. Study Design and Treatment

This was a randomized, open-label, two-period, two-sequence, and crossover-designed bioequivalence study. The number of subjects needed for this study was initially estimated to be 26 subjects, based on the intra-subject coefficient of variance (CV) of pramipexole obtained from the literature [11]. At the end of the study, the required minimum number of subjects was recalculated based on the actual CV obtained from this study, in order to meet the statistical power of 80%. The actual number of subjects that can be evaluated for pharmacokinetic analysis determines the adequacy of the actual study power.

On the first sampling day of the first study period, subjects were given either the test or the reference formulation with 200 mL of plain water after an overnight fast and a pre-dose blood sampling. They could have their standardized lunch and dinner 4 h and 10 h, respectively, after their first dosing. No xanthine-containing food or beverages and fruit juices were allowed 24 h before and during the entire sampling days.

Blood samplings, using drawing needle 22G (Vacuette Visio Plus, Nipro Medical Industries, Osaka, Japan) and EDTA vacuum tubes (Vacuette, Greiner Bio-One Ltd., Amata Nakorn, Thailand) or disposable syringes (Terumo, Terumo Corporation, Binan, -Philippines), were performed at 20 and 40 min, and 1, 1.5, 2, 2.5, 3, 4, 6, 8, 12, 16, 24, 36, and 48 h after drug administration. The collection of blood samples at each time point was centrifuged (Centrifuge EBA 320, Hettich Zentrifugen, Andreas Hettich GmbH & Co. KG, Tuttlingen, Germany) at 1538 relative centrifugal force (RCF) for 15 min to separate the plasma. The plasma of each sample was then transferred to a clean tube and stored in a freezer of −20 °C ± 5 °C until assayed. The same procedure was repeated in the second study period with the alternate drug after a one-week washout period.

### 2.4. Bioanalytical Method and Pharmacokinetic Analysis

#### 2.4.1. Materials

The materials and chemicals used to prepare, treat, and analyze the bio-samples in the study consisted of acetonitrile (Liquid chromatography Grade, PT Merck Chemicals and Life Sciences, Jakarta, Indonesia), formic acid (PT Merck Chemicals and Life Sciences), blank plasma (Indonesia Red Cross, Jakarta, Indonesia), ammonia solution (PT Merck Chemicals and Life Sciences), dichloromethane (Analytical Grade, PT Merck Chemicals and Life Sciences), ammonium formate (Sigma Aldrich Pte Ltd., Singapore, Singapore), and 2-propanol (Analytical Grade, PT Merck Chemicals and Life Sciences).

#### 2.4.2. Validated Bioanalytical Method for Quantification of the Drug Concentration

Plasma samples were quantified for pramipexole concentration using a validated ultra performance liquid chromatography with mass spectrometry (UPLC-MS/MS) detector (Waters, Acquity^®^ H-Class system with Xevo^®^ TQD Detector, Waters Corporation, Milford, MA, USA) in positive ion electrospray ionization mode, using a multiple reaction monitoring (MRM) method and atenolol as the internal standard (IS). The transitions used were 212.1 → 153.1 for pramipexole and 267.1 → 190.1 for the IS. The UPLC-MS/MS was equipped with an Acquity UPLC^®^ BEH Amide (1.7 µm, 2.1 × 50 mm) analytical column. Formic acid with a 0.1% concentration in a mixture of water and acetonitrile (15:85 v/v) as a mobile phase was pumped at a rate of 0.4 mL/min through the column. Before being injected into the chromatography system, the plasma sample was treated according the sample extraction procedure as described below. The validated condition of the UPLC-MS/MS system as indicated by its adequate sensitivity, specificity, linearity, accuracy, and precision (both within and between days) is presented in Table 1.

#### 2.4.3. Sample Extraction

Each plasma sample was dispensed in an appropriate tube, and the atenolol solution was then added. The content of the tube was vortexed (Thermolyne Vortex Mixer, Barnstead Thermolyne Corporation, Dubuque, IA, USA) and centrifuged, and NH_4_OH and dichloromethane solvent were then added. The mixture was vortexed for another 1 min and centrifuged for 10 min at 4500 rpm. The organic layer was transferred to another tube and evaporated to dryness under nitrogen (N_2_) at 50 °C. The residue was subsequently reconstituted by acetonitrile and then vortexed for 30 s. The solution was transferred into the vial insert and injected into the UPLC-MS/MS system that had been set at an appropriate condition previously described.

#### 2.4.4. Pharmacokinetic Data Analysis

The maximum plasma concentration (C_max_) and time to reach C_max_ (t_max_) was directly obtained from the observed data. The area under the plasma concentration curve from time zero to the last observed measurable concentration (AUC_0-t_) was calculated by the trapezoidal method, while the area under the plasma concentration curve extrapolated to infinite time (AUC_0-∞_) was calculated as the sum of AUC_0-t_ plus C_t_/λ_z_. C_t_ was the last observed measurable concentration, and λ_z_ was the terminal rate constant, determined as the slope of linear regression of the log-transformed concentration–time curve [12]. The plasma concentration half-life (t_1/2_) was determined as 0.693/λ_z_ [12].

The pharmacokinetic parameters of pramipexole in this study was statistically evaluated via an analysis of variance (ANOVA) after the transformation of the data to their logarithmic values, using Phoenix^®^ WinNonlin^®^ 6.3 (Certara, L.P., St. Louis, MO, USA). The statistical comparison of t_max_ and t_1/2_ between both formulations were analyzed on the original data by Wilcoxon matched-pairs test and Student’s paired *t*-test, respectively. The 90% confidence intervals (CIs) of the geometric means of the test/reference ratios for AUC and C_max_ were between 80.00% and 125.00% to meet the acceptance criteria for bioequivalence [12,13].

#### 2.4.5. Incurred Sample Reanalysis

In this study, the incurred sample reanalysis (ISR) was performed in compliance with the European Medicines Agency (EMA) Guideline on bioanalytical method validation [14].

## 3. Results

### 3.1. Study Population

Twenty-six healthy adult male and female subjects were invited to undergo the eligibility assessments at the time of screening. Of them, three subjects did not come for any of the study periods; thus, there were only 23 eligible subjects recruited and enrolled in the study. Of the enrolled subjects, one subject dropped out during the first period of the study due to nausea and vomiting, and 22 subjects remained available for pharmacokinetic data analysis. All subjects were healthy Indonesians and consisted of eight males and fourteen females aged 18–50 years old. They also met all eligibility criteria of the study. Baseline characteristics of the study population are described in Table 2.

### 3.2. Pharmacokinetic and Statistical Analysis

**Figure 1:**
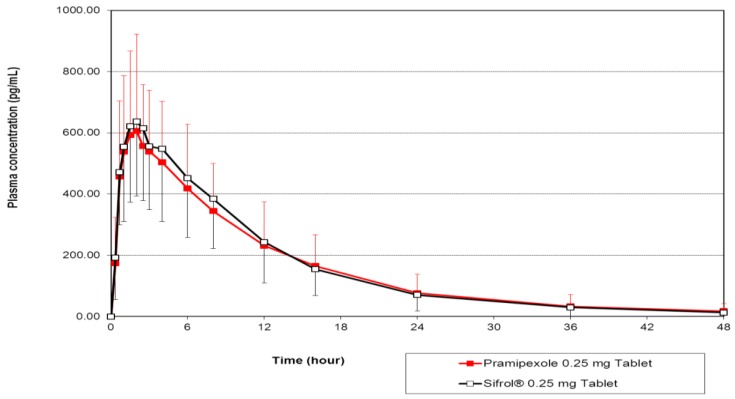
The mean plasma concentration–time profiles of pramipexole in 22 subjects after a single dose oral administration of 0.25 mg pramipexole tablets produced by PT Dexa Medica and the reference drug, Sifrol^®^, produced by Boehringer Ingelheim Pharma GmbH & Co. KG.

Figure 1 shows the plot of mean pramipexole concentrations in 22 evaluable subjects after a single dose oral administration of the test and reference formulations. Detailed mean values of pramipexole pharmacokinetic parameters in addition to their bioequivalence analyses evaluated as 90% CIs of the geometric means of the test/reference ratios for the AUC and C_max_ are presented in Table 3.

### 3.3. Safety and Tolerability

Among the enrolled 23 study subjects, one subject experienced nausea and vomiting in the first period, resulting in dropout. This subject received the test formulation in that first period. Other adverse events reported from those who received the test formulation included syncope (one subject) and nausea (one subject), while one subject who received the reference formulation reported headache in the first period of study. All of the above-mentioned adverse events were rated as mild and resolved at the end of study. No serious adverse event was encountered.

### 3.4. Incurred Sample Reanalysis

The ISR of study samples was performed in separate runs at different days. Of all subjects who completed the study, seven subjects were randomly selected for ISR. The reanalysis sample points were also selected randomly from blood sampling time-points around C_max_ (three samples) and the elimination phase (two samples), for each study period. Therefore, a total of 70 repeat samples were included in the ISR.

The EMA Guideline requires that the concentration obtained for the initial analysis and the concentration obtained by reanalysis should be within +20% of their mean for at least 67% of the repeats [14]. The requirement was also fulfilled in this study. Of 70 samples, 48 samples (68.57%) met the ISR criteria.

## 4. Discussion

The present study was conducted following the generally accepted design for bioequivalence testing [12,15], with a single-dose of pramipexole. The crossover design was the best design option to remove between-subject variability. The test and reference formulations of pramipexole were administered in a randomized manner. The code was prepared using a permuted block allocation and the table of random numbers from Dixon & Massey [16]. Although pramipexole may be administered without regard to meals, a fasting state was enforced. The administration of pramipexole with food is known to delay the rate of its absorption by about one hour [5,6]. A fasting state was believed to better prevent the possibility of high inter- and intra-subject variability in the rate and extent of drug absorption.

Healthy human subjects with restricted body mass index were enrolled in the study in order to maintain the same total dose across study subjects. Using the intra-subject CV of pramipexole from the literature [11], the sample size was estimated by means of confidence intervals (CIs) as tabulated by Diletti et al. [17]. There were only 23 subjects who were finally enrolled in the study. Of them, only 22 subjects were evaluable for pharmacokinetic analysis, with the previously mentioned justification. This number was then re-evaluated based on the actual intra-subject CV for pramipexole AUC_0-t_ of this study, i.e., 10.67% (Table 3). Based on that CV, the use of 22 subjects was adequate to draw a statistical conclusion with the power of no less than 90%. The intra-subject CV of pramipexole obtained from this study was approximately similar to those found in the literature [11,18,19].

The total number of sampling times and the selected time-points in this study were sufficient to characterize the blood level profile of pramipexole. The sampling times were also extended to more than three times the plasma concentration half-life of pramipexole to provide a sufficient elimination period of the drug. A one-week washout period was also considered adequate to eliminate the administered dose from the body. An adequate washout period is important to ensure equal residual effects, one of the crucial assumptions in the two-period crossover design.

According to the Code of Federal Regulations Title 21 (21 CFR) 320.1 of the Food and Drug Administration (FDA), bioequivalence is defined as the absence of a significant difference in the rate at which, or the extent to which, the active ingredient or active moiety in pharmaceutical equivalents or pharmaceutical alternatives becomes available at the site of drug action when administered at the same molar dose under similar conditions in an appropriately designed study [12]. The AUC_0-t_ and AUC_0-∞_ represent the proportionality to the extent of drug absorbed and eliminated from the body, while the C_max_ and t_max_ estimate the rate of drug absorption [15]. From the result of the study, 90% CIs of the geometric means of pramipexole test/reference ratios for AUC and C_max_ lay between 80.00% and 125.00%; thus, bioequivalence was concluded. Statistical comparison of t_max_ of the test and reference formulations of the 0.25 mg pramipexole tablets showed an insignificant difference. It was also observed that the t_max_ median of both formulations corresponded to data from the literature, which states that pramipexole is rapidly absorbed from the gastrointestinal tract and reaches its peak plasma concentrations within about 2 h in fasting patients [6]. The t_1/2_ mean (SD) of the test and reference pramipexole formulations were 8.82 (4.02) h and 8.83 (3.95) h, respectively. Those values were not significantly different, demonstrating a comparable rate of drug elimination from the body.

The concepts of bioequivalence have been considered important in the last three decades when approving generic drugs [20]. With this concept, drug regulators require that generic formulations should have equivalent clinical effects with its innovator counterparts, resulting in interchangeability of both products. In compliance with the requirement, we have also conducted bioequivalence studies on several generic formulations [21,22,23,24]. Our current study on pramipexole was carried out with the same requirements in mind. Although similar bioequivalence studies on different formulations of pramipexole have been conducted [11,18,19], the present study still needed to be executed since the result of one bioequivalence study is specific only for one particular product and cannot be extrapolated or generalized for other generic products of the same active substance.

The availability of generic drugs will benefit patients because they are less expensive than the innovators’ drugs. Generic drugs are cheaper than their branded counterparts because manufacturers do not need to conduct costly clinical trials to investigate the safety and efficacy of the generic version of a drug that has already been proven to be safe and effective for years. Bioequivalence studies attempt to prove that two formulations work in the same way for the same amount of time in the body, which then indicates therapeutic equivalence between both formulations [12,15]. Our present study demonstrated that the 0.25 mg pramipexole tablet produced by PT Dexa Medica, as a generic formulation is bioequivalent to its reference. Therefore, based on the result of the present bioequivalence study, including observation on the adverse events encountered during the study, the test pramipexole tablet is regarded to have equivalent safety and clinical efficacy to that of the innovator’s product used as the reference in this study.

## 5. Conclusions

It was concluded that the two formulations of the 0.25 mg pramipexole tablets evaluated in this study were bioequivalent.

## Figures and Tables

**Table 1:** Validation data of the analytical method used for the determination of pramipexole in human plasma via ultra performance liquid chromatography with mass spectrometry (UPLC-MS/MS), using atenolol solution as the internal standard (IS).

Parameters		Low (60.32 pg/mL)	At concentration of	
			Medium (1005.40 pg/mL)	High (1759.45 pg/mL)
Precision ^a^	intra-assay	4.55%	4.57%	3.74%
	inter-assay	7.30%	5.13%	5.29%
Accuracy ^a^	intra-assay	0.65%	−6.74%	−3.81%
	inter-assay	−0.68%	−7.81%	−6.83%
Stability	at −20°C (stable until 44 days)	−3.81% to +0.49%	–	–3.56% to +7.45%
	at room temperature (stable until 6 h)	−14.60% to +4.49%	–	−12.41% to −3.51%
	freeze and thaw (stable until 4 cycles)	−11.01% to +6.84%	–	−12.87% to +3.83%
Linearity: the linearity of the standard calibration curves was obtained (*r* of 0.9974 on day 1, 0.9975 on day 2, and 0.9998 on day 3).				
LLOQ: the LLOQ has been established at 20.12 pg/mL.				
Selectivity: The % diff of the analyte and internal standard interferences ranged from 2.71%–17.92% and 0.01%–0.03%, respectively. From the result, it can be concluded that there were no interferences of the analyte and internal standard compounds.				
Range: the range of quantification was established as 20.12–2011.60 pg/mL.				

^a^ shown by the difference of the measured values to actual values (% diff). The acceptance limit for coefficient of variation (CV) lies within 15% for all concentrations in intra-assay and inter-assay precision as well as accuracy determinations. LLOQ, lower limit of quantitation.

**Table 2:** Baseline characteristics of the study population.

Subject	Gender	Age (Years)	Weight (kg)	Height (m)	BMI (kg/m^2^)	Smoking Status (Cigarettes/Day) ^a^
1	F	18	51.5	1.52	22.29	-
2	M	34	43	1.54	18.13	3
3	F	49	57	1.54	24.03	-
4	F	46	43	1.50	19.11	-
5	F	46	60	1.55	24.97	-
6	M	18	65	1.75	21.22	6
7	F	32	54.5	1.64	20.26	-
8	F	29	51	1.54	21.50	-
9	F	42	54	1.47	24.99	-
10	F	38	59	1.54	24.88	-
11	F	23	61	1.57	24.75	-
12	F	42	48	1.50	21.33	-
13	F	29	51	1.56	20.96	-
14	F	44	56	1.50	24.89	-
15	M	36	64	1.60	25.00	8
16	F	50	60	1.57	24.34	-
17	M	23	55	1.63	20.70	6
18	M	23	52	1.65	19.10	8
19	M	46	45	1.56	18.49	-
20	M	26	61	1.69	21.36	3
21	M	25	48	1.58	19.23	6
22	M	28	49	1.63	18.44	5
23	F	24	57	1.57	23.12	-
Mean	-	33.52	54.13	1.57	21.87	-
SD	-	10.37	6.35	0.07	2.46	-
Min	-	18	43	1.47	18.13	-
Max	-	50	65	1.75	25.00	-
%CV	-	30.93%	11.73%	4.23%	11.26%	-

^a^ smoking status is defined as the amount of cigarettes consumed per day. BMI: body mass index; SD: standard deviation; CV: coefficient of variance.

**Table 3:** Pharmacokinetic parameters and bioequivalence evaluation of pramipexole in 22 subjects after a single-dose oral administration of 0.25 mg pramipexole tablets of both test (T) and reference (R) formulations.

Parameter	Test Formulation Mean (SD)	Reference Formulation Mean (SD)	Geometric Mean Ratio of T/R (90% CI) ^a^	*p* Values	%CV
AUC_0-t_ (pg·h/mL) ^b^	7355.76 (3793.14)	7542.56 (3492.79)	95.89% (90.73%–101.34%)	0.2053 ^b^	10.67%
AUC_0-∞_ (pg·h/mL) ^b^	7996.24 (3861.00)	8196.72 (3555.44)	95.53% (89.75%–101.68%)	0.2208 ^b^	12.05%
C_max_ (pg/mL) ^b^	684.43 (308.51)	727.41 (251.07)	92.11% (84.35%–100.58%)	0.1228 ^b^	17.05%
t_1/2_ (h)	8.82 (4.02)	8.83 (3.95)	-	NS ^d^	-
t_max_ (h) ^c^	2.00 (0.67–4.00)	1.75 (0.67–3.00)	-	NS ^e^	-

^a^ bioequivalence criteria are defined as 90% confidence intervals (CIs) of the geometric means of the test/reference (T/R) ratios lies between 80.00% and 125.00% for AUC_0-t_, AUC_0-∞_, and C_max_; ^b^ statistical calculations by analysis of variance (ANOVA) for AUC and C_max_ were based on ln-transformed data; ^c^ the values are expressed in median (range); ^d^ analysis was performed by Student’s paired *t*-test; ^e^ analysis was performed by Wilcoxon matched pair test. SD: standard deviation; %CV: mean intra-subject variability.
